# EGFR‐mutated stage IV non‐small cell lung cancer: What is the role of radiotherapy combined with TKI?

**DOI:** 10.1002/cam4.4192

**Published:** 2021-08-10

**Authors:** Bailong Liu, Hui Liu, Yunfei Ma, Qiuhui Ding, Min Zhang, Xinliang Liu, Min Liu

**Affiliations:** ^1^ Department of Radiation Oncology The First Hospital of Jilin University Changchun China

**Keywords:** EGFR mutation, radiotherapy, TKI

## Abstract

Lung cancer is the leading cause of cancer‐related death globally and poses a considerable threat to public health. Asia has the highest prevalence of epidermal growth factor receptor (EGFR) mutations in patients with non‐small cell lung cancer (NSCLC). Despite the reasonable response and prolonged survival associated with EGFR‐tyrosine kinase inhibitor (TKI) therapy, the acquisition of resistance to TKIs remains a major challenge. Additionally, patients with EGFR mutations are at a substantially higher risk of brain metastasis compared with those harboring wild‐type EGFR. The role of radiotherapy (RT) in EGFR‐mutated (EGFRm) stage IV NSCLC requires clarification, especially with the advent of next‐generation TKIs, which are more potent and exhibit greater central nervous system activity. In particular, the feasible application of RT, including the timing, site, dose, fraction, and combination with TKI, merits further investigation. This review focuses on these key issues, and provides a flow diagram with proposed treatment options for metastatic EGFRm NSCLC, aiming to provide guidance for clinical practice.

## Graphical abstract

For the oligometastatic setting, radiotherapy (RT) to both primary and metastatic lesions might prolong both progression‐free survival and OS. Reduced‐dose thoracic RT and critical restriction of the mean lung dose before tyrosine kinase inhibitor (TKI) resistance onset hold some promise. Although epidermal growth factor receptor (EGFR) TKIs can exert synergistic effects with RT, this regimen should be applied with caution owing to the risk of adverse effects, especially lung damage. In the first‐ or second‐generation EGFR TKI era, upfront brain RT showed better efficacy; however, this is expected to change with the advent of next‐generation TKIs with increased central nervous system efficacy. The likely beneficiaries of upfront brain RT, especially stereotactic radiosurgery, with the arrival of the new drugs should be identified through clinical trials.

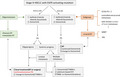

## 1 INTRODUCTION

Lung cancer has the highest morbidity and mortality.^1^ Epidermal growth factor receptor (EGFR) mutations are more prevalent in Asian population than in non‐Asian patients,^2^, ^3^ which are more likely to have brain metastasis than wild‐type counterpart. ^4^, ^5^ In China, EGFR mutations comprise the largest proportion of driver mutations in lung adenocarcinoma, with a frequency of 63.1%,^6^ substantially higher than the 17% observed in the USA.^7^ EGFR tyrosine kinase inhibitors (TKIs) are the standard first‐line treatment option for advanced or metastatic non‐small cell lung carcinoma (NSCLC) patients harboring TKI‐sensitive EGFR mutations, and confer a greater progression‐free survival (PFS) benefit than treatment with cytotoxic chemotherapy.^8^, ^9^, ^10^, ^11^, ^12^ Compared with its wild‐type counterpart, EGFRm NSCLC is more aggressive and has a higher likelihood of metastasizing to the brain and bone.^13^ The resistance to TKI remains inevitable and the role of radiotherapy (RT) in this population has been underestimated yet. The survival benefit brought by RT to both the primary tumor and oligometastatic sites, the critical issues such as RT dose, time, means which can maximize the tumor control and minimize the treatment toxicity need more high‐quality and evidence‐based guidance. Besides, the central nervous system (CNS) failure is a prominent problem in oncogene‐driven NSCLC entity. With the advent of brand‐new CNS‐active TKI, the role of brain RT will be challenged. Therefore, a systemic review of the most beneficiary population and the reasonable means of RT is illustrative.

## 2 FAILURE PATTERN OF ADVANCED EGFRm NSCLC AFTER TKI

The failure pattern after TKI exposure included solitary (primary or another site) and multiple lesion progression. Oya et al. reviewed 181 patients with advanced EGFRm NSCLC with initial TKI failure (gefitinib, erlotinib, and afatinib). The failure pattern included solitary lesion progression in 73 patients (40%) and multiple lesion progression in 108 patients (60%). The T790M mutation (87/181, 48%) was more prevalent in patients with solitary lesion progression than in those with multiple lesion progression (58% vs. 24%; *p* < 0.0001).^14^ Yoshida et al. reported that 104 patients developed progressive disease (PD) after an initial response to EGFR‐TKI treatment. Twenty‐four patients (23%) harbored solitary failure while 80 (77%) developed multiple lesions.^15^ Brain metastases, EGFR mutation type, and TP53 status were the main determinants of TKI response and survival.^16^ Therefore, thoracic RT (TRT) and brain RT are two main critical issues in dealing with the RT role of EGFRm stage IV NSCLC combined with TKI.

## 3 TRT COMBINED WITH TKI

Chen MJ et al. reviewed 316 stage III/IV NSCLC patients treated with gefitinib between October 2002 and September 2011. The median PFS was 238 days and the common failure sites were the lungs (62.34%), followed by bone (17.72%), the CNS (16.14%), and the liver (9.49%). Single‐site progression accounted for most of the cases (81.01%).^17^ Similarly, Tang et al. reported that 41.25% of patients (105 in total; 14 stage IIIB and 91 stage IV; >5 metastases: 44) treated with EGFR TKIs experienced original site failure.^18^ This highlighted that, for locally advanced/metastatic EGFRm NSCLC, it is critical to strengthen local control, especially at the primary lung lesion, which will greatly influence the survival.

### 3.1 Upfront TRT with TKI

In 2019, Zheng and colleagues conducted a single‐arm study of upfront TRT concurrently with first‐line EGFR TKI (gefitinib: 1, erlotinib: 9) in 10 stage IV NSCLC patients with limited metastatic lesions (≤10) harboring TKI‐sensitive EGFR mutations (*n* = 4 exon 21 L858R mutation; *n* = 6 exon 19 deletions). TRT was performed within 2 weeks of EGFR TKI application (54–60 Gy/27–30 F/5.5–6 w). The normal lung irradiation dose was strictly limited (mean lung dose [MLD] = 5.25 ± 3.75 Gy, V20 = 12 ± 4%). The median time to progression in the irradiated site (iTTP) was 20.5 months, significantly longer than the 8.4–13.1 months normally required for the onset of resistance to first‐generation EGFR TKIs. Compared with the ENSURE study,^19^ this combination yielded a numerically higher 1‐year PFS rate (57.1% vs. 43%) and longer PFS (median: 13.0 vs. 11.0 months). However, 20% of the patients developed grade ≥3 radiation pneumonitis (RP) (including 1 with grade 5 RP),^20^ indicating that, even with the strict restriction of the normal lung dose, caution is needed when performing TRT concurrently with first‐line EGFR TKI owing to the relatively high incidence of RP.

### 3.2 TRT applied before TKI resistance onset

Tang et al. retrospectively reviewed 105 stage IIIB/IV EGFRm NSCLC patients treated with TKIs. Eighty patients exhibited disease progression before the cut‐off date, and could be classified into three groups: those with original site progression (*n* = 33, 41.25%); those with new distant site progression (*n* = 34, 42.5%); and those with both (*n* = 13, 16.25%). Additionally, exon 21 mutation was closely linked with original site failure. The median time to response to TKI was 2 months. Therefore, the authors proposed that the optimal time for TRT intervention might be after this time point and before the onset of secondary resistance to TKIs.^18^ This paradigm is reasonable because the primary pulmonary tumor shrank after TKI exposure and the radiation field was smaller, allowing dose escalation to the gross tumor volume and avoiding severe damage to adjacent normal organs. Additionally, TKI efficacy can be correctly evaluated as TRT is not initially performed.

In 2018, Xu and colleagues conducted a relatively large‐scale retrospective study on the role of consolidative local ablative therapy (LAT) in oligometastatic (≤5 metastases) stage IV EGFRm NSCLC without progression after first‐line EGFR TKI treatment. The 145 enrolled patients were divided into 3 groups: an all‐LAT group (*n* = 51), in which LAT was applied to both the primary tumor and metastatic sites; a part‐LAT group (*n* = 55); and a non‐LAT group (*n* = 39). Patients in the all‐LAT group displayed a markedly improved median PFS compared with those in the part‐LAT (20.6 vs. 15.6 months, *p* < 0.001) and non‐LAT (20.6 vs. 13.9 months, *p* < 0.001) groups. The median overall survival (OS) in the all‐LAT group was also superior to that of both the part‐LAT (40.9 vs. 34.1 months, *p* = 0.009) and non‐LAT (40.9 vs. 30.8 months, *p* < 0.001) groups. There was no difference in either PFS or OS between the part‐LAT and non‐LAT groups. However, after propensity score matching to balance the baseline features (more advanced T/N stage in the part‐LAT and non‐LAT groups), the all‐LAT group also achieved a survival advantage (PFS/OS). Multivariate analysis confirmed that LAT to primary tumor was associated with better PFS (HR = 0.36, *p* < 0.001) and OS (HR = 0.45, *p* < 0.001). LAT to the primary tumor included surgery (9.2%), stereotactic radiosurgery (SRS) (16.9%), and external beam radiation therapy (EBRT) (55–63 Gy) (73.9%). In the non‐LAT group (*n* = 39), 25 patients (64.1%) underwent salvage LAT after disease progression. This highlighted that deferral of LAT can impair survival.^21^

Similarly, in a retrospective study conducted by Hu et al., 231 lung adenocarcinoma patients with oligometastatic lesions (1 organ, ≤5 lesions) and EGFR mutation were divided into 2 groups, 1 receiving first‐generation EGFR TKI monotherapy (*n* = 88) and the other local consolidative therapy (LCT) plus TKI before progression (*n* = 143). The addition of LCT conferred benefits for both PFS (15 vs. 10 months, *p* = 0.000) and OS (34 vs. 21 months, *p* = 0.001).^22^ The NCT03916913 clinical trial has been launched to evaluate the efficacy and toxicity of local RT at all EGFRm NSCLC disease sites, including primary lung tumors and oligometastatic lesions (≤3), in patients who did not experience disease progression after at least 3 months of TKI therapy.

### 3.3 TRT applied at the time of oligo‐progression of thoracic lesions

Despite the good response to TKIs in patients with advanced and metastatic NSCLC harboring TKI‐sensitive EGFR mutations, the PFS associated with first‐generation EGFR TKI therapy (gefitinib, erlotinib) is only 8.0–13.7 months.^11^, ^19^, ^23^, ^24^, ^25^, ^26^, ^27^ Local progression due to acquired resistance is common. Wang Y and colleagues retrospectively reviewed 44 NSCLC patients with active EGFR mutations who developed local progression (defined as 1–3 lesions in one organ) after front‐line first‐generation TKI therapy. These patients underwent concurrent local RT to 50 lesions with prior TKI. The addition of RT brought both TTP and PFS benefits (TTP1+TTP2 vs. TTP1: 21.7 vs. 16.0 months, *p* = 0.01; PFS1+PFS2 vs. PFS1: 21.3 vs. 16.0 months, *p* = 0.027). Additionally, non‐smokers over the age of 56 benefited substantially from local RT. The subgroups with performance status (PS) 0–1 or 2 attained prolonged median TTP from local RT.^28^

### 3.4 The lung damage caused by TRT combined with TKIs

One of the above mentioned 44 patients who received RT (50–60 Gy/5–30 F for pulmonary lesions) to local progression lesions with upfront EGFR TKI developed grade ≥3 RP.^28^

Zhuang et al. analyzed 24 stage IIIA–IV NSCLC patients who underwent erlotinib therapy concurrent with TRT (46–66 Gy). The incidence of grade ≥2 RP (*n* = 9) was 37.5%, including 16.7% grade 2 (*n* = 4), 8.3% grade 3 (*n* = 2), and 12.5% grade 5 RP (*n* = 3). However, the lung dose parameters of the three patients who died of RP were acceptable (V20: 20%, 26%, and 26%; V5: 47%, 55%, and 67%; MLD: 1209 cGy, 1446 cGy, 1453 cGy). This highlighted that erlotinib increases the risk of RP, in agreement with several reports that have indicated that erlotinib can exert adverse effects on pulmonary interstitium. Additionally, the authors reported that the RP‐related threshold values of V5, V10, V15, V20, and V30 were >44%, >29%, >27%, >22%, and >17%, respectively, while the MLD was >1027 cGy.^29^, ^30^, ^31^, ^32^

The third‐generation EGFR TKI, osimertinib, has been reported to induce an especially high rate of RP (grade ≥2) in patients with TRT combined simultaneously with osimertinib, despite low lung V5, V20, and MLD values.^33^

Overall, the first‐line EGFR TKI therapy followed by TRT before the onset of secondary resistance to TKI showed better efficacies in patients with oligometastatic EGFRm NSCLC (Table 1), but whether this could bring survival benefits need to be verified by large‐scaled prospective randomized trials. Because of the higher radiosensitivity of EGFRm tumors, the TRT dose can be appropriately reduced, thereby decreasing the RP risk. Caution is warranted when administering TRT combined with different TKIs (first, second, third, and next generation) owing to the risk of lung damage, and strict restriction of the normal lung dose is necessary. Patient treatment should be individualized according to the specific mutation subtype. Patients with mutations in exon 21 might benefit more from TRT as they are at higher risk of original site failure. Ongoing clinical trials should help to clarify the role of TRT in EGFRm advanced/stage IV NSCLC (Table 2).

**TABLE 1:** Published studies of TRT combined with TKI for EGFR‐mutated stage IV NSCLC

Study	Published year/PMID	Country	Study nature	Design	TKI	Outcome
Positive results						
Zheng L et al.	2019/31040256^20^	China	Single‐arm, phase II study	*N* = 10 Upfront TRT concurrently with TKI for EGFRm stage IV NSCLC with limited metastatic lesions (≤10) (exon 21 L858R mutation: 4; exon 19 deletion: 6)	Erlotinib and gefitinib	iTTP: 20.5 months; 1‐year PFS rate (57.1%); and median PFS (13.0 m). 20% grade ≥3 RP (including 1 grade 5)
Xu Q et al.	2018/29852232^21^	China	Retrospective, single‐institution	*N* = 145 Oligometastatic stage IV EGFRm NSCLC (≤5 metastases) without progression after first‐line EGFR TKI treatment. All‐LAT group (*n* = 51, LAT to both primary tumor and metastatic sites), part‐LAT group (*n* = 55), and non‐LAT group (*n* = 39)	Erlotinib, gefitinib, and icotinib	Median PFS: all‐LAT versus part‐LAT (20.6 vs. 15.6 months, *p* < 0.001); all‐LAT versus non‐LAT (20.6 vs. 13.9 months, *p* < 0.001). Median OS: all‐LAT versus part‐LAT (40.9 vs. 34.1 months, *p* = 0.009); all‐LAT versus non‐LAT (40.9 vs. 30.8 months, *p* < 0.001). No PFS or OS benefit: part‐LAT versus non‐LAT. Multivariate analysis: primary tumor LAT –better PFS (HR =0.36, *p* < 0.001) and OS (HR =0.45, *p* < 0.001)
Hu F et al.	2019/30341018^22^	China	Retrospective, single‐institution	*N* = 231 EGFRm NSCLC with oligometastatic lesions (1 organ, ≤5 lesions). Group 1: first‐generation EGFR TKI alone (*n* = 88); Group 2: LCT plus TKI before progression (*n* = 143)	First‐generation EGFR TKI	The addition of LCT attained both PFS (15 vs. 10 m, *p* = 0.000) and OS (34 vs. 21 m, *p* = 0.001) benefits, respectively

Abbreviations: EGFR, epidermal growth factor receptor; EGFRm, epidermal growth factor receptor‐mutated; LAT, local ablative therapy; LCT, local consolidative therapy; NSCLC, non‐small cell lung cancer; PFS, progression‐free survival; RP, radiation pneumonitis; TKI, tyrosine kinase inhibitor; TRT, thoracic radiotherapy.

**TABLE 2:** Ongoing clinical trials of RT to primary tumor in EGFR‐mutated advanced/stage IV NSCLC

Trial ID	Name	Country	Estimated enrollment	Start date	Estimated primary completion date	Estimated study completion date	Arm	Primary outcome measure
NCT03916913	TKI followed by thoracic radiotherapy for stage IV EGFR‐mutant NSCLC	China	*N* = 85	2019/1	2022/1	2023/1	Local RT on all sites of disease including primary and metastatic lesions for EGFRm oligometastatic NSCLC (≤3 metastatic lesions) without disease progression after ≥3 months of TKI therapy	PFS
NCT03667820	Phase II trial of osimertinib in combination with SABR in EGFR‐mutant advanced NSCLC	USA	*N* = 37	2018/9	2021/4	2022/4	Osimertinib 8 weeks SABR for residual lesion then osimertinib. SABR to progression site when possible	PFS
NCT03074864	Intercalated combination of erlotinib and radiotherapy for patients with EGFR‐mutant, unresectable, locally advanced NSCLC	China	*N* = 90	2017/2	2019/6	2020/6	Erlotinib 150 mg/day for 12 weeks then local RT followed by 24‐week erlotinib maintenance	ORR
NCT01553942	Afatinib sequenced with concurrent chemotherapy and radiation in EGFR‐mutant non‐small cell lung tumors: The ASCENT trial	USA	*N* = 30	2012/4	2020/12	2021/12	Stage IIIA EGFRm NSCLC: afatinib for two 4‐week cycles; concurrent radiation and chemotherapy with cisplatin/pemetrexed for two 3‐week cycles; Surgery; Adjuvant chemotherapy; Consolidation with afatinib for 2 years for participants who responded to induction afatinib	ORR
NCT03410043	Randomized phase II trial of LCT after osimertinib for patients with EGFR‐mutant metastatic NSCLC	USA	*N* = 143	2018/1	2022/1	2023/1	Stage IIIB/IV or recurrent EGFRm NSCLC Group I (LCT): osimertinib PO QD for 6–12 weeks. Then surgery and/or RT, continue osimertinib during and after radiation therapy. Group II (no LCT): osimertinib PO QD	PFS

Abbreviations: EGFR, epidermal growth factor receptor; EGFRm, epidermal growth factor receptor‐mutated; LCT, local consolidative therapy; NSCLC, non‐small cell lung cancer; ORR, objective response rate; PFS, progression‐free survival; PO QD, Latin abbreviation 'peros, quaque die', means 'orally, once daily'; RT, radiotherapy; SABR, stereotactic ablative radiation; TKI, tyrosine kinase inhibitor.

## 4 BRAIN RT COMBINED WITH TKI

### 4.1 CNS failure in EGFRm NSCLC patients

Central nervous system failure is a frequent complication for EGFR‐driven NSCLC patients. In the EGFR‐TKI era, the causes of NSCLC‐related death have changed. More NSCLC patients with EGFR mutations die of CNS progression than those harboring wild‐type EGFR (44.8% vs. 8.3%, *p* < 0.001).^34^ This might be explained by the poor ability of TKIs to cross the blood–brain barrier (BBB), the intrinsic resistance of metastatic clones, longer survival, and the higher tendency of EGFRm patients to develop brain metastases (BM).^35^, ^36^

In the BRAIN clinical trial, treatment‐naïve EGFRm NSCLC patients with BM of ≥3 lesions were randomized into two groups, one receiving icotinib alone and the other whole‐brain irradiation (WBI; 30 Gy/10 F) plus concurrent or sequential chemotherapy. Icotinib alone yielded a markedly improved median intracranial PFS (iPFS) compared with WBI+chemotherapy (10.0 vs. 4.8 months, *p* = 0.014).^37^ Although WBI+TKI was not used as the control group, these results clearly indicated that TKIs should be the first‐choice management modality for oncogene‐driven NSCLC with BM, while RT (WBI/SRS/SRT) should be delayed until intracranial progression, especially in patients without obvious CNS symptoms.

Overall, first‐generation EGFR TKIs are poorly BBB‐permeable, which greatly limits their efficacy in reducing the CNS tumor burden. Gefitinib and erlotinib CSF penetration rates are just 1.13 ± 0.36% and 2.77 ± 0.45%, respectively.^38^ Icotinib, at a dose of 375 mg tid, attained a median CSF penetration rate of 6.1%,^39^ while the CNS penetration rate of 40 mg/day afatinib on day 8 was 2.45 ± 2.91%.^40^ *K*_puu,brain_ (unbound brain‐to‐plasma drug concentration ratio) represents a reliable predictor of BBB permeability, with value >0.3 indicative of good diffusion across the BBB.^41^ *K*_puu,brain_ in preclinical animal models for gefitinib, erlotinib, afatinib, osimertinib, and AZD3759 was 0.02, 0.11, 0.007, 0.39, and 1.3, respectively.^42^, ^43^ Third‐ and new‐generation EGFR TKIs have greater BBB permeability,^43^ which will influence the option, timing, and means of cranial RT application.^44^ The results of AURA3, a randomized phase III trial, indicated that osimertinib monotherapy had greater CNS efficacy than pemetrexed/platinum chemotherapy in treating EGFR T790M‐positive, TKI‐resistant NSCLC after prior TKI (median CNS PFS: 11.7 vs. 5.6 months, *p* = 0.004).^45^ Osimertinib elicited a satisfactory intracranial objective response rate (ORR) of 54% and a disease control rate (DCR) of 92% in T790M‐positive NSCLC patients (*n* = 50) who progressed after prior EGFR TKI intervention.^46^

Recently, a meta‐analysis was conducted of 12 retrospective studies comprising 1553 EGFRm patients with BM at initial diagnosis. The addition of brain RT greatly prolonged OS (*p* < 0.001) and iPFS (*p* < 0.001) compared with TKI treatment alone.^47^ In an era of ever‐improving systemic treatment strategies, two major challenges remain. First, for TKI‐naïve EGFRm patients with asymptomatic BM, should brain RT be upfront or withheld until intracranial failure, and which is the optimal cranial RT module (whole‐brain radiotherapy [WBRT], SRS, or WBRT with a simultaneous integrated boost [SIB])? Second, up to now, most researches were based on first‐ or second‐generation EGFR TKIs with limited permeability of BBB. With the advent of third‐/next‐generation TKI harboring better CNS efficacy, what is the role of brain RT?

### 4.2 The ideal timing for brain RT

Another single‐institution retrospective study enrolled 64 EGFRm NSCLC patients with BM. Thirty‐five patients underwent first‐line TKI with concurrent cranial RT including SRS, WBRT, or WBRT‐SIB as determined by the radiation oncologist. Twenty‐nine patients used TKI alone. All the patients took first‐generation EGFR TKIs (erlotinib, gefitinib, or icotinib). Upfront definitive brain RT conferred both OS (31 vs. 24 months, *p* = 0.019) and iPFS benefits (25 vs. 16 months; *p* = 0.019).^48^ Saida et al. conducted a retrospective analysis of 104 EGFRm NSCLC patients with BM from 10 institutions. Thirty‐nine of the patients underwent upfront brain RT, including SRS or SRT (*n* = 19), WBRT (*n* = 16), and both WBRT+SRS or SRT (*n* = 4). Sixty‐five patients used a first‐ or second‐generation TKI. Despite patients in the RT group displaying a greater number of symptomatic and larger BMs, upfront RT still prolonged the time to treatment failure (TTF) compared with the TKI‐only treatment (11.2 vs. 6.8 months, *p* = 0.038). However, TTF, CNS‐PFS, and OS were similar between the upfront SRS and WBRT treatment groups.^49^ Chen YH reviewed 134 treatment‐naïve EGFRm NSCLC patients who received first‐ or second‐generation EGFR TKIs (erlotinib: 49; gefitinib: 62; and afatinib: 23). Thirty‐eight (29.1%) of these patients underwent brain RT before disease progression, and achieved significant improvements in iPFS.^50^

Additionally, another small‐scale study analyzed 78 EGFRm lung adenocarcinoma patients with BM and categorized them into 2 groups, namely, a TKI+RT group (*n* = 49, including 23 patients with asymptomatic BM; 35 WBRT, 14 SRS) and a TKI‐only group (*n* = 29, including 22 patients with asymptomatic BM). Upfront brain RT greatly prolonged iPFS (21.5 vs. 15 months, *p* = 0.036). In the cohort with asymptomatic BM, compared with TKI monotherapy, RT+TKI treatment elicited a superior median iPFS (21.5 vs. 14.8 months, *p* = 0.026) and OS (36 vs. 23 months, *p* = 0.041).^51^ Wang et al. analyzed 132 EGFRm NSCLC patients with asymptomatic BM who all used first‐generation TKIs. Upfront or concurrent RT (*n* = 46) conferred improved OS when compared with upfront TKI (*n* = 86) (24.9 vs. 17.4 months, *p* = 0.035) despite the higher proportion of ≥4 intracranial lesions in the upfront or concurrent RT group (57.8% vs. 39.8%, *p* = 0.035). Additionally, 74 patients with asymptomatic BM who underwent brain RT (WBRT: 63, SRS: 11) were grouped into 3 cohorts: upfront RT (*n* = 13), concurrent RT (*n* = 33), and RT after TKI (*n* = 28). The iPFS and OS of the three groups were 11.3, 11.1, and 8.1 months (*p* = 0.032) and 26.2, 21.9, and 17.1 months (*p* = 0.085), respectively.^52^ These findings indicated that the deferral of cranial RT led to an inferior prognosis, even in the population with asymptomatic BM.

In contrast, in 2016, Byeon et al. performed a single‐institution, retrospective study on 121 newly diagnosed EGFRm NSCLC patients with BM. Patients in Group A (*n* = 59) underwent brain RT followed by TKI (SRS: 32, WBRT: 26, and both: 1) while those in Group B (*n* = 62) received TKI alone. The patients took first‐generation TKIs (gefitinib: 103; erlotinib: 18). Upfront cranial RT did not confer any benefit for 3‐year OS (A vs. B: 71.9% vs. 68.2%, *p* = 0.678), iPFS (A vs. B: 16.6 vs. 21.0 months, *p* = 0.492), or extracranial PFS (A vs. B: 12.9 vs. 15.0 months, *p* = 0.77), but did improve the intracranial DCR (79.7% vs. 59.7%, *p* = 0.019). However, the interval of brain evaluation in the two groups was not equal, which inevitably affected the accuracy of the iPFS data.^53^

He et al. reviewed 104 treatment‐naïve EGFRm NSCLC patients with BM. Fifty‐six patients underwent concurrent WBRT (30 Gy/10 F/2 w) and first‐generation TKI while 48 took a TKI alone, including 20 who received salvage WBRT after developing resistance to TKIs. The addition of concurrent WBRT greatly improved iPFS compared with TKI monotherapy (17.7 vs. 11.0 months, *p* = 0.015); however, OS was unchanged. The number of BM was closely associated with iPFS (>3 vs. ≤3: 12.5 vs. 18.0 months, *p* = 0.044). Upfront WBRT prolonged the iPFS of patients with >3 BM (17.6 vs. 9.2 months, *p* = 0.001), but not that of patients with ≤3 BM (19.2 vs. 14.5 months, *p* = 0.526).^54^

### 4.3 The ideal means of brain RT

#### 4.3.1 WBRT

WBRT can disrupt the BBB, and is expected to increase the CNS permeability of systemic drugs. Chen et al. evaluated 141 NSCLC patients with BM harboring TKI‐sensitive EGFR mutations. All the patients received EGFR TKIs, while 94 (66.7%) also received WBRT. After a median follow‐up of 20.3 months, the addition of WBRT markedly improved the OS compared with TKI treatment alone (median OS: 14.3 vs. 2.3 months, 1‐year OS: 81.9% vs. 59.6%, *p* = 0.002) despite the presence of more unfavorable baseline features in the WBRT arm, such a greater number of BM (*p* = 0.043), larger BM (*p* = 0.046), and neurological symptoms (*p* = 0.005). Multivariate analysis also suggested that WBRT was an independent prognostic factor for prolonged OS. Among this study cohort, 3.5% used the third‐generation TKI, osimertinib.^35^Another small‐sample retrospective study documented 139 EGFRm NSCLC patients with BM. Seventy‐nine used a first‐generation TKI alone, while 60 received TKI plus WBRT (30 Gy/10 F). The RT group included 10 patients who underwent delayed RT for intracranial progression after TKI while others underwent simultaneous WBRT for BM >3 cm or symptomatic BM. The addition of WBRT contributed to a prolonged median intracranial TTP (30.0 vs. 18.2 months, *p* = 0.001), but not to OS (48.0 vs. 41.1 months, *p* = 0.912).^55^

Similarly, Chen YS and colleagues undertook a single‐institution retrospective study of 132 EGFRm NSCLC patients with BM. The patients were divided into two groups—an EGFR‐TKI with concomitant WBRT group (*n* = 53) and a TKI‐only group (*n* = 79). The addition of upfront WBRT (30 Gy/10 F) improved both the intracranial ORR (67.9% vs. 39.2%, *p* = 0.001) and median iTTP (24.7 vs. 18.2 months, *p* = 0.004), but not OS (48.0 vs. 41.1 months, *p* = 0.74). Primary WBRT manifested iTTP superiority for patients with symptomatic BM (27.0 vs. 18.2 months, *p* = 0.008) but not for those with asymptomatic BM (24.7 vs. 20.0 months, *p* = 0.193). In multivariate analysis for iTTP, WBRT was the only independent favorable prognostic factor (*p* = 0.004). Additionally, a longer iTTP (exceeding 22 months) was associated with markedly improved OS (58.0 vs. 28.0 months, *p* = 0.001). This highlighted that the effective control of cranial lesions could be converted into an improvement in OS.^56^

The initial WBRT in such a molecularly selected entity was questioned mainly for its neurological toxicity. However, the uncontrolled intracranial tumor deteriorated the normal neurological functions. Li et al. proposed that satisfactory intracranial DCR gained by RT avoided the neurological deterioration potently.^57^ Therefore, the optimal timing of WBRT needs to be identified by further prospective trials especially with the application of CNS‐active EGFR TKIs.

#### 4.3.2 SRS

Lee et al. reviewed 198 EGFRm NSCLC patients with BM. The patients were divided into four groups according to the timing and type of RT applied, that is, an immediate WBRT group (*n* = 75), an immediate SRS group (*n* = 21), a delayed RT until intracranial progression group (DRT, *n* = 27), and a no brain RT group (NRT, *n* = 75). The median OS of the four groups was 18.5, 55.7, 21.1, and 18.2 months, respectively (*p* = 0.008), and the median PFS was 6.9, 14.0, 7.9, and 8.5 months, respectively (*p* = 0.001). Immediate SRS elicited the best OS and PFS effects, whereas WBRT failed. However, these findings were unreliable owing to the unbalanced baseline features. Patients in the immediate SRS group had the fewest lesions, both intracranially and extracranially. In this study, the 27 patients (13.6%) who had received EGFR T790M inhibitor treatment survived markedly longer than the other 171 patients who did not (41.1 vs. 19.8 months, *p* < 0.001).^44^

In 2017, Magnuson et al. conducted a retrospective study of TKI‐naïve EGFR‐mutant NSCLC patients with BM (*n* = 351, 6 institutions). These patients were categorized into three groups based on the timing and type of RT: an upfront SRS followed by TKI group (*n* = 100), an upfront WBRT followed by TKI group (*n* = 120), and a primary TKI followed by WBRT or SRS for intracranial progression group (*n* = 131). A total of 98% of the patients used erlotinib (*n* = 344). At baseline, the upfront WBRT arm had more unfavorable prognostic factors (*p* = 0.001), while the other two groups shared similar characteristics. Upfront RT (SRS or WBRT) conferred a clear OS benefit compared with the primary TKI setting. The OS of the three groups was 46, 30, and 25 months, respectively (*p* < 0.001). This survival benefit conveyed by upfront RT was most prominent in the favorable prognosis subgroup (GPA 2–3.5). Moreover, a multivariable analysis indicated that upfront SRS or WBRT was closely linked with a lower probability of intracranial failure (*p* = 0.062 and *p* = 0.64, respectively). The median iPFS for the initial SRS, WBRT, and TKI arms was 23, 24, and 17 months, respectively (*p* = 0.025).^58^Wang et al. evaluated 49 TKI‐naïve EGFRm NSCLC patients who developed BM. Forty received WBRT while five underwent SRS. All the patients received a first‐generation TKI (icotinib: 37, gefitinib: 9, and erlotinib: 3). SRS prolonged the median OS compared with WBRT (37.7 vs. 21.1 months, *p* = 0.194).^52^

In a retrospective study involving 145 patients, Xu et al. reported that consolidative LAT to brain metastasis improved the median OS for patients with oligometastatic stage IV EGFRm NSCLC without progression after initial EGFR TKI (38.2 vs. 29.2 months, HR = 0.48, *p* = 0.002). The LAT for brain metastasis included WBRT (*n* = 17), SRS (*n* = 27), and surgery+WBRT (*n* = 5).^21^

### 4.4 The population that benefits the most from upfront brain RT

For patients with multiple BM and with lung‐molGPA 2.5–4.0, upfront whole‐brain RT brought significantly prolonged iPFS (12.8 vs. 10.1 months, *p* = 0.014) and OS (23.3 vs. 15.3 months, *p* = 0.005) when compared with the TKI‐resistant group. This study did not include osimertinib.^48^ In 2018, Wang CY et al. reported the results of a meta‐analysis of seven retrospective studies (WBRT: 2; SRS/WBRT: 5) involving 1086 patients. Upfront brain RT was defined as the application of RT initiated before, or within 4 weeks, of EGFR TKI therapy. The control group in the seven studies received first‐generation TKI. The addition of upfront brain RT prolonged both iPFS (*p* = 0.028) and OS (*p* = 0.015). Notably, for the limited BM setting (≤3 BM), upfront RT exerted OS superiority (HR = 0.54, *p* = 0.000), while for patients with >3 BM, RT failed to show OS advantage.^59^ Liu and colleagues reviewed 113 EGFRm NSCLC patients with BM. Forty‐nine patients underwent upfront brain RT (WBRT: 37; SRS: 12) within 4 weeks after TKI initiation, while 64 received TKI alone, including 27 who underwent salvage brain RT (WBRT: 22; SRS: 5) for BM progression. Upfront brain RT conferred iPFS superiority (21.4 vs. 15 months, *p* = 0.001) but without OS benefit. Furthermore, for the population with DS‐GPA 0–2, early brain RT prolonged OS (*p* = 0.025).^60^ Miyawaki et al. reported that treatment‐naïve EGFRm NSCLC patients with limited BM (1–4) showed improved OS from upfront SRS for BM.^61^

Zhu et al. evaluated the role of upfront brain RT in 133 patients. Sixty‐seven of these patients received TKI plus RT (WBRT: 63; SRS: 4), while 66 took TKI alone (erlotinib or gefitinib). For the subgroup of patients with exon 21 mutations, TKI+RT improved both OS (22.0 vs. 13.5 months, *p* = 0.004) and iPFS (14.0 vs. 9.5 months, *p* = 0.001) compared with TKI monotherapy. However, for the subgroup of patients with exon 19 mutations, there was no difference in either OS or iPFS between the two arms.^62^

### 4.5 Third‐generation EGFR TKIs and brain RT

FLAURA Asian subset analysis revealed that osimertinib exhibits CNS activity. Compared with first‐generation TKIs (gefitinib: 250 mg/day, erlotinib: 150 mg/day), 80 mg/day osimertinib improved both PFS (16.5 vs. 11.0 months, HR = 0.54, *p* < 0.0001) and CNS PFS (not calculable vs. 13.8 months, HR = 0.55, *p* = 0.118) in patients with EGFRm advanced NSCLC (2). In the BLOOM study, osimertinib (160 mg/day) showed meaningful therapeutic efficacy in the CNS as well as manageable safety.^63^

In a retrospective, single‐institution study conducted by Xie and colleagues, 40 EGFRm NSCLC patients with BM and treated with osimertinib were divided into 3 groups (A–C). Group A comprised patients with progressing BM who received osimertinib alone (*n* = 11); Group B consisted of patients with progressing BM who underwent RT when starting osimertinib (*n* = 9); and In Group C, patients had stable BM and were treated with osimertinib alone (*n* = 20). RT before starting osimertinib failed to prolong TTF, PFS, or OS. This indicated that, to minimize the risks associated with radiation‐related toxicity, delaying radiation may be an option for some patients with EGFRm NSCLC with BM who initially respond to osimertinib.^64^ Many new EGFR TKIs have potent CNS‐penetrating ability and exhibit satisfactory CNS response rates of 40%–70%.^65^ Therefore, upfront WBRT might be safely postponed once highly CNS‐active EGFR TKIs become available.^66^

In 2019, an international clinical trial (OUTRUN; NCT03497767) was initiated to clarify whether upfront SRS plus osimertinib can improve intracranial disease control for EGFRm NSCLC patients with ≤10 de novo or developed brain metastases after first‐generation EGFR TKI therapy compared with osimertinib treatment alone. This trial excluded cases with leptomeningeal or brain stem metastasis. Another trial, NCT03769103, is aimed at evaluating upfront SRS (1–5 fractions) followed by osimertinib 80 mg daily for treatment‐naïve EGFRm NSCLC patients with ≤10 brain metastases.

Upfront cranial RT seems to show better efficacy, but whether it can improve the intracranial DCR and prolong the iPFS or OS for EGFRm NSCLC patients with BM treated with first‐ or second‐generation EGFR TKIs needs further study. Several reports also support that upfront brain RT improves both iPFS and OS for patients with asymptomatic BM. SRS might be a reasonable RT approach for prolonging OS and preserving neurological function (Table 3). The population that is most likely to benefit from upfront brain RT should be identified through randomized multicenter prospective clinical trials. Retrospective studies have shown that subgroups, such as those comprising patients with exon 21 mutations, controlled extracranial metastases, and a limited number of BM might benefit more from upfront cranial RT, especially SRS. With the advent of EGFR TKIs with greater CNS activity, brain RT might be deferred to prolong survival and improve quality of life. The subgroups of EGFRm NSCLC patients with BM treated with these new TKIs that will be suitable for upfront brain RT should be determined by national clinical trials (Table 4).

**TABLE 3:** Published studies of brain RT combined with TKI for EGFR‐mutated NSCLC with BM

Study	Published year/PMID	Country	Study nature	Design	TKI	Outcome
Positive results						
Zeng YD et al.	2012/22631670^102^	China	Retrospective, single‐institution	*N* = 90 A: gefitinib+WBRT (*n* = 45); B: gefitinib alone (*n* = 45)	Gefitinib	A versus B: BM ORR: 64.4% versus 26.7%, *p* < 0.001; BM DCR: 71.1% versus 42.2%, *p* = 0.006; median time to progression of BM: 10.6 versus 6.57 months, *p* < 0.001; median OS: 23.4 versus 14.83 months, *p* = 0.002
Chen YS et al.	2016/27627582^56^	China	Retrospective, single‐institution	*N* = 132 A: EGFR‐TKI plus concomitant WBRT (*n* = 53) B: TKI alone (*n* = 79)	Gefitinib Erlotinib	A versus B: Intracranial ORR: 67.9% versus 39.2%, *p* = 0.001; median iTTP: 24.7 versus 18.2 months, *p* = 0.004; OS: 48.0 versus 41.1 months, *p* = 0.74
Zhu QQ et al.	2017/28076323^62^	China	Retrospective, 2 institutions	*N* = 133 *N* = 67: TKI+RT (WBRT: 63; SRS: 4) *N* = 66: TKI alone	Erlotinib Gefitinib	Exon 21 mutations: TKI+RT: better OS (22.0 vs. 13.5 months, *p* = 0.004) and iPFS (14.0 vs. 9.5 months, *p* = 0.001). Exon 19 mutations: no difference
Magnuson WJ et al.	2017/28113019^58^	USA	Retrospective, multi‐institutional (6 institutions)	*N* = 351 Upfront SRS followed by TKI (*n* = 100); upfront WBRT followed by TKI (*n* = 120); and primary TKI followed by WBRT or SRS for intracranial progression (*n* = 131)	98% Erlotinib (*n* = 344)	OS: upfront SRS versus upfront WBRT versus TKI followed by RT (46 vs. 30 vs. 25 months, *p* < 0.001). upfront RT: more benefit for favorable prognosis subgroup (GPA 2–3.5)
Liu YM et al.	2017/29340055^60^	China	Retrospective, single‐institution	*N* = 113 Early brain RT+TKI: *n* = 49; TKI+salvage brain RT: *n* = 27; TKI alone: *n* = 37	Erlotinib, gefitinib, and icotinib	Superior IC‐PFS: early brain RT versus without early brain RT (21.4 vs. 15.0 months, *p* = 0.001); no OS difference. DS‐GPA: 0–2, early brain RT is independent factor of improved OS, *p* = 0.025
Wang WX et al.	2018/30393484^52^	China	Retrospective, single‐institution	*N* = 45 symptomatic: WBRT: 40; SRS: 5 *N* = 132 asymptomatic: upfront or concurrent RT: 46; upfront TKI: 86 Further analysis *N* = 74 Upfront (U) RT: 13 Concurrent (C) RT: 33 RT after TKI (R): 28	Erlotinib, gefitinib, and icotinib	Symptomatic: Improved median OS (SRS vs. WBRT: 37.7 vs. 21.1 months, *p* = 0.194). Asymptomatic: upfront or concurrent RT: improved OS 24.9 versus 17.4 m (*p* = 0.035) compared with upfront TKI iPFS U versus C versus R—11.3 versus 11.1 versus 8.1 months, *p* = 0.032. OS U versus C versus R—26.2 versus 21.9 versus 17.1 months, *p* = 0.085
Sung S et al.	2018/29644484^103^	Korea	Retrospective, single‐institution	*N* = 81 TKI+RT: *n* = 40 (WBRT: 21; SRS: 19); TKI alone: *n* = 41	Gefitinib and erlotinib	2‐year intracranial progression: TKI plus RT group versus TKI alone group (36.5% vs. 62.2%, *p* = 0.006). No difference in neurological death or OS
Ke SB et al.	2018/30536070^55^	China	Retrospective, single‐institution	*N* = 139 WBRT+TKI: *n* = 60, including delayed WBRT *n* = 10; TKI alone: *n* = 79	Erlotinib and gefitinib	TKI+WBRT: improved iTTP (median 30.0 vs. 18.2 months, *p* = 0.001), but no OS benefit
Chen H et al.	2018/30383657^100^	China	Retrospective, single‐institution	*N* = 105 Group A: EGFR‐TKIs alone (*n* = 39); Group B: EGFR‐TKIs+concurrent WBRT (*n* = 34); Group C: WBRT followed by EGFR‐TKIs (*n* = 32)	Erlotinib, gefitinib, and icotinib	Intracranial ORR for Groups A, B, and C was 66.7%, 85.3%, and 75%, respectively (*p* < 0.05). The median intracranial PFS for Groups A, B, and C was 6.8, 12.4, and 9.1 months, respectively (*p* < 0.05)
Chen Y et al.	2019/31399067^51^	China	Retrospective, single‐institution	*N* = 78 Brain RT+TKI (*n* = 49, 23 asymptomatic; 35 WBRT, 14 SRS); TKI (*n* = 29, 22 asymptomatic)	Erlotinib, gefitinib, and icotinib	miPFS RT+TKIs versus TKIs: 21.5 versus 15 months, *p* = 0.036. Asymptomatic BM miPFS: RT+TKIs versus TKIs: 21.5 versus 14.8 months, *p* = 0.026; OS: 36 versus 23 months, *p* = 0.041
Saida Y et al.	2019/31507098^49^	Japan	Retrospective, 10 institutions	*N* = 104 Upfront TKI (*n* = 65); Upfront RT (*n* = 390; SRS or SRT: 19, WBRT: 16, both WBRT and SRS or SRT: 4)	Gefitinib, erlotinib, and afatinib	TTF: upfront RT versus upfront TKI (11.2 vs. 6.8 months, *p* = 0.038)
An N et al.	2019/31632080^101^	China	Retrospective, single‐institution	*N* = 64 TKI+RT (*n* = 35; RT = WBRT/SRS/WBRT‐SIB); TKI (*n* = 29)	Erlotinib, gefitinib, and icotinib	TKI+RT: improved OS (31 vs. 24 months, *p* = 0.019) and iPFS (25 vs. 16 months; *p* = 0.019)
Chen CH et al.	2019/31370314^35^	China (Taiwan)	Retrospective, single‐institution	*N* = 141 TKI+WBRT (*n* = 94); TKI (*n* = 47)	Afatinib: 17; erlotinib: 75; gefitinib: 97; and osimertinib: 5	Median OS: TKI+WBRT versus TKI (14.3 vs. 2.3 months). 1‐year OS rate: TKI+WBRT versus TKI (81.9% vs. 59.6%, *p* = 0.002)
He ZY et al.	2019/30936745^54^	China	Retrospective, single‐institution	*N* = 104 TKI+WBRT (*n* = 56); TKI (*n* = 48; 20 salvage WBRT upon BM progression)	Erlotinib, gefitinib, and icotinib	Median iPFS: TKI+WBRT versus TKI (17.7 vs. 11 months, *p* = 0.015); no OS difference. Subgroup analysis: TKI+WBRT improved iPFS in patients with >3 BM, *p* = 0.001; no iPFS difference in patients with ≤3 BM, *p* = 0.526
Zhao L et al.	2020/31892337^48^	China	Retrospective, single‐institution	*N* = 344 (BM ≥4) WBRT TKI‐naïve (*n* = 207); WBRT TKI‐resistant (*n* = 137)	No osimertinib	Lung‐mol GPA 2.5–4 WBRT TKI naïve: better iPFS (12.8 vs. 10.1 months, *p* = 0.014) and OS (23.3 vs. 15.3 months, *p* = 0.005)
Negative results						
Byeon S et al.	2016/27447711^53^	Korea	Retrospective, single‐institution	*N* = 121 A: Brain RT followed by TKI (*n* = 59: SRS: 32, WBRT: 26, both: 1); B: TKI alone (*n* = 62)	Gefitinib Erlotinib	A versus B 3‐year OS (71.9% vs. 68.2%, *p* = 0.678); iPFS (16.6 vs. 21.0 months, *p* = 0.492); extracranial PFS (12.9 vs. 15.0 months, *p* = 0.77); intracranial DCR: (79.7% vs. 59.7%, *p* = 0.019). A: WBRT versus SRS: 3‐year OS, iPFS no difference; SRS longer extracranial PFS

Abbreviations: DCR, disease control rate; EGFR, epidermal growth factor receptor; iPFS, intracranial progression‐free survival; NSCLC, non‐small cell lung cancer; ORR, objective response rate; RT, radiotherapy; SRS, stereotactic radiosurgery; SRT, stereotactic RT; TKI, tyrosine kinase inhibitor.

**TABLE 4:** Ongoing clinical trials of RT in EGFR‐mutated NSCLC with BM

Trial ID	Name	Country	Estimated enrollment	Start date	Estimated primary completion date	Estimated study completion date	Arm	Primary outcome measure
NCT01763385	Erlotinib with concurrent brain radiotherapy and secondary brain radiotherapy after recurrence with erlotinib in NSCLC non‐increased intracranial pressure symptomatic brain metastases (TRACTS)	China	*N* = 210 Randomized	2012/11	2016/5	2016/5	Erlotinib and secondary brain radiotherapy––erlotinib until brain tumor progression, then given brain radiotherapy, and continued to take erlotinib until extracranial lesion progression. Erlotinib and concurrent brain radiotherapy––erlotinib with concurrent brain radiotherapy, and continued to take erlotinib after radiotherapy until recurrence or termination for other reasons	OS
NCT03497767	A randomised phase II trial of osimertinib with or without SRS for EGFR‐mutated NSCLC with brain metastases (OUTRUN)	International Trans‐Tasman Radiation Oncology Group	*N* = 80 Randomized	2019/8	2021/9	2022/3	Arm A: 80 mg osimertinib taken once daily. Arm B: upfront SRS followed by 80 mg osimertinib taken once daily	Intracranial progression‐free survival at 12 months
NCT03769103	Study of osimertinib+SRS versus osimertinib alone for brain metastases in EGFR‐positive patients with NSCLC	International	*N* = 76 Randomized	2019/3	2025/4	2025/4	Arm A: 80 mg osimertinib taken once daily Arm B: Upfront SRS (1–5 F) followed by 80 mg osimertinib taken once daily	Intracranial progression‐free survival at 12 months

Abbreviations: EGFR, epidermal growth factor receptor; NSCLC, non‐small cell lung cancer; RT, radiotherapy; SRS, stereotactic radiosurgery.

**FIGURE 1:**
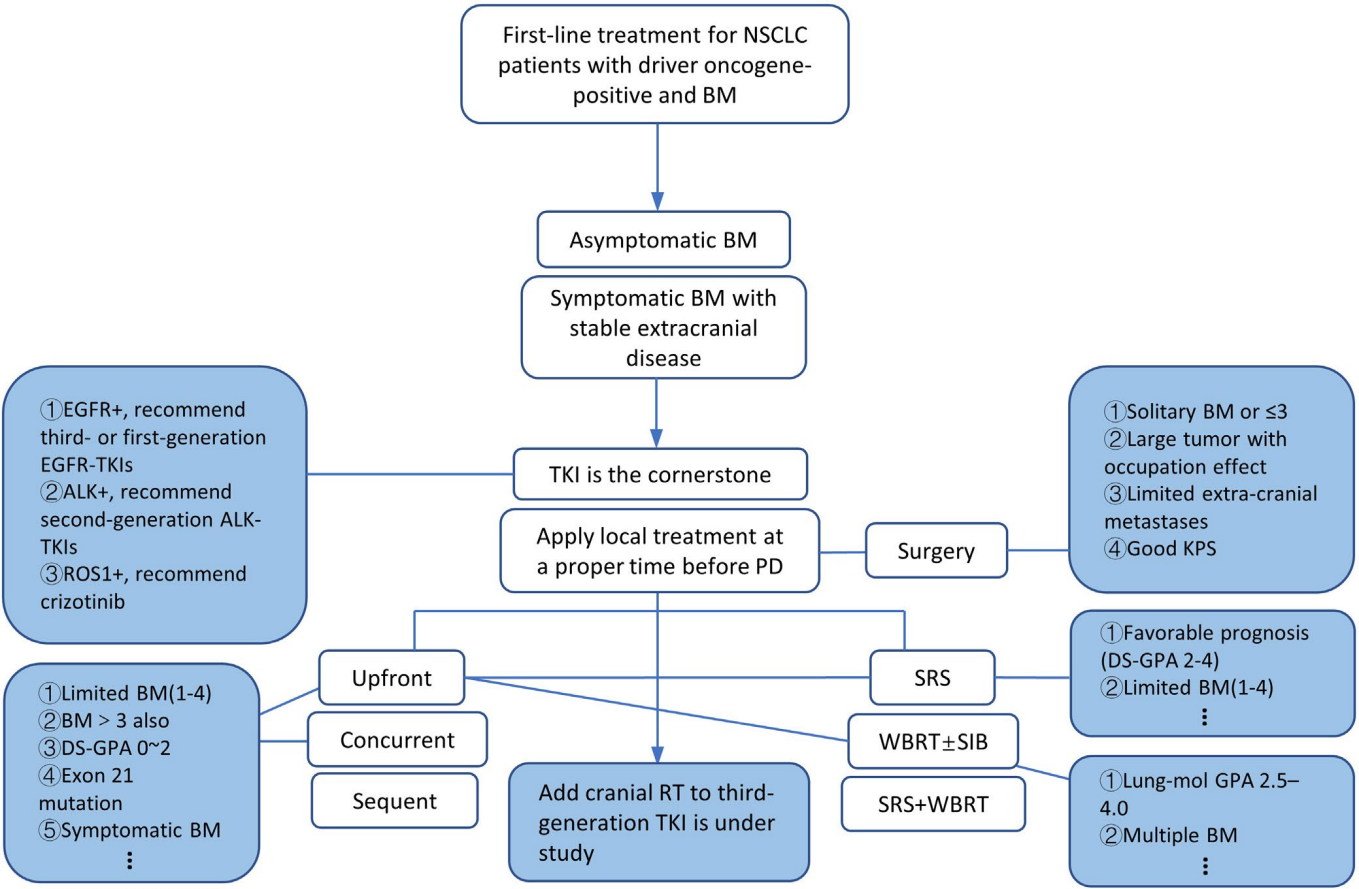
Preliminary treatment outlines for driver oncogene‐positive NSCLC with brain metastases. ALK, anaplastic lymphoma kinase; BM, brain metastasis; EGFR, epidermal growth factor receptor; NSCLC, non‐small cell lung cancer; PD, progressive disease; ROS1, c‐Ros oncogene 1 receptor tyrosine kinase; SIB, simultaneous integrated boost; SRS, stereotactic radiosurgery; TKI, tyrosine kinase inhibitor; WBRT, whole‐brain radiotherapy

**FIGURE 2:**
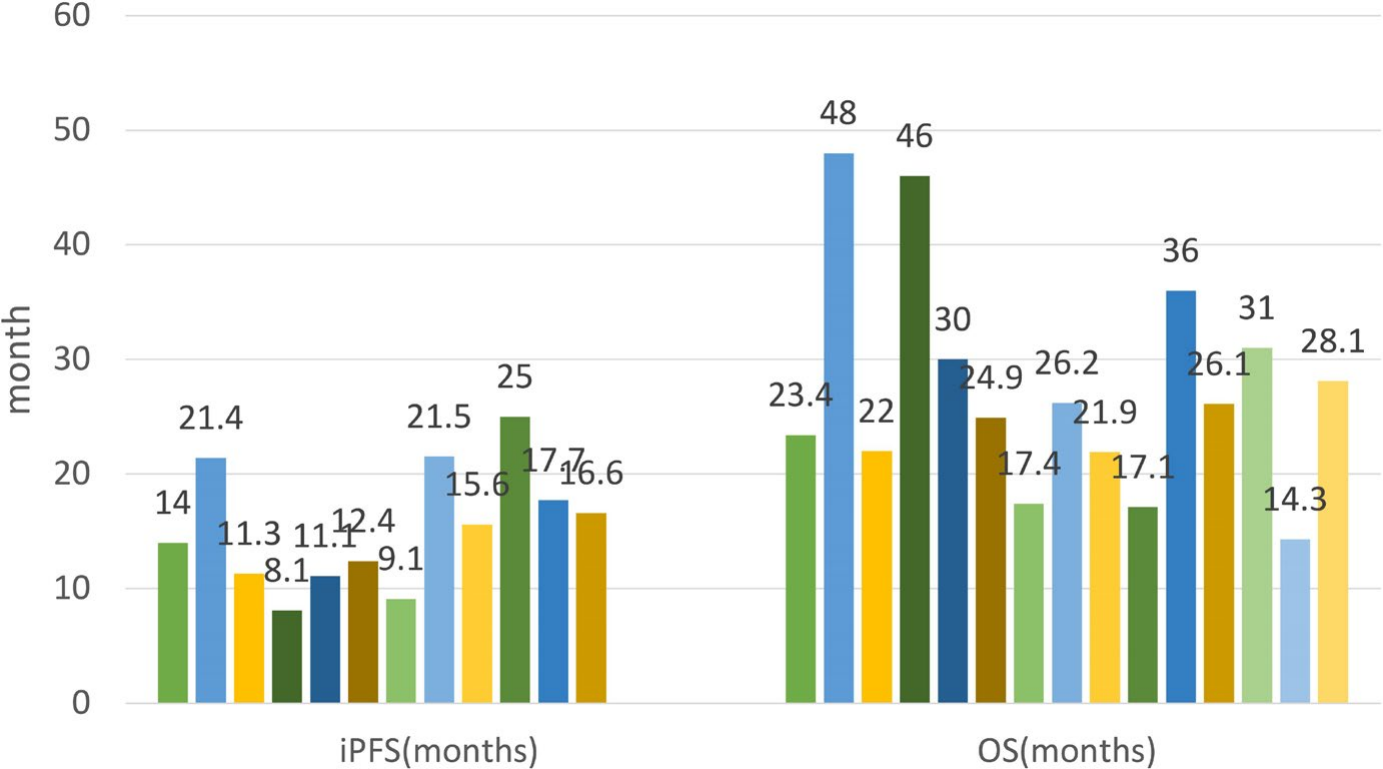
Pooled survival results from retrospective studies in this review mentioned about the effect of first‐ or second‐generation EGFR‐TKI combined with cranial RT before progression. iPFS ranges from 8.1 to 25 months,^49^, ^51^, ^52^, ^53^, ^54^, ^60^, ^62^, ^100^, ^101^ and OS ranges from 14.3 to 48 months.^35^, ^49^, ^51^, ^52^, ^54^, ^56^, ^58^, ^62^, ^102^ EGFR: epidermal growth factor receptor; iPFS, intracranial progression‐free survival; RT, radiotherapy; TKI, tyrosine kinase inhibitor

Overall, for this special BM population, treatment is complex because various clinical situations need to be considered. At present, for those asymptomatic or symptomatic BM with stable extracranial disease, on the basis of TKI, early use of local RT before progression could be recommended in clinical practice. We provide an initial treatment outline for reference in Figures 1 and 2.

## 5 RT TO SPINE/BONE METASTASES

Anand et al. evaluated the efficacy of hypo‐fractionated stereotactic body RT in spinal metastasis with or without epidural extension in 52 patients with 76 lesions, preferably oligo‐metastasis. Of these, 20 patients (34.8%) had malignant epidural compression (MEC). Most of the primaries were breast cancer (30.8%) and lung cancer (19.2%). One‐year local control and overall survival were 94% and 68%, respectively. Complete pain relief was seen in 90% of patients with MEC and 93.75% without MEC (P = NS) and 60% of patients in both groups had achieved neurological improvement. Acute side effects were generally mild and no long‐term complications were observed.^67^

Chang et al. observed a significant survival benefit from early addition of multiple‐target metastatic RT after systemic disease controlled during TKI treatment in 25 TKI‐sensitive stage IIIB/IV NSCLC patients (9 with oligo‐metastases, 10 patients with bone metastases, 40%), with an ORR of 84.0%, median PFS of 16.0 months, and 3‐year survival rate of 62.5%.^68^

Another multi‐institutional respective study found that the median OS in patients with stage IV NSCLC including oligo‐metastases treated with SRT (stereotactic RT) (SRT for 27% bone metastasis) concomitant to TKI was 23 months, and both univariate and multivariate analyses showed that SRT and TKI duration were independent prognostic factors for OS.^69^

A prospective phase II trial reported the long‐term results of radical treatment (surgery or RT) for subgroup of NSCLC patients with synchronous oligo‐metastases (18% bone; all bone metastases were treated with RT: 54 Gy in 30 twice‐daily fractions of 1.8 Gy). The primary endpoint OS was 13.5 months, comparable with other metastatic locations of adrenal and brain (*p* = 0.52). The 1‐, 2‐, and 3‐year OS were 56.4%, 23.3%, and 17.5%, respectively. The secondary endpoint PFS was 12.1 months. The 1‐year PFS was 51.3%. This indicated that radical therapy of NSCLC patients with synchronous oligo‐metastases was associated with long‐term PFS.^70^

## 6 RT TO OTHER METASTATIC SITES WITH TKI

Xu et al. evaluated the effect of consolidative LAT on oligometastatic stage IV EGFRm NSCLC without progression after first‐line EGFR TKI, and confirmed that patients with adrenal metastases gained an OS benefit from LAT (37.1 vs. 29.2 months, HR = 0.48, *p* = 0.032). The LAT to adrenal metastasis included surgery (*n* = 13, 54.2%), SRS (*n* = 5, 20.8%), and EBRT (45–50 Gy) (*n* = 6, 25%).^21^

Elamin et al. explored the role of LCT in metastatic EGFRm NSCLC after initial TKI. A total of 129 patients took first‐line TKI alone while 12 underwent LCT plus TKI treatment (oligo‐metastasis with ≤3 metastases: 8; with >3 metastases: 4). The LCT regimen included SBRT or hyper‐fractionated RT for 11 patients and surgery for 1 patient. The addition of LCT conferred a greatly improved PFS compared with TKI treatment alone (36 vs. 14 months, *p* = 0.0024). One of the 11 patients received RT to left iliac bone metastasis and 1 underwent RT to all 3 bilateral pulmonary nodules.^71^ Hu et al. further confirmed that LCT conferred a survival benefit for patients with oligometastatic EGFRm NSCLC, an effect that was observed regardless of oligometastatic sites or mutation subtype.^22^

## 7 THE POTENTIAL MECHANISM UNDERLYING THE SYNTHETIC KILLING EFFECT EXERTED BY RT AND TKI

### 7.1 The EGFRm subtype exhibits greater radiosensitivity

Das et al. revealed that NSCLC cell lines that harbored ionizing radiation‐sensitive mutations in EGFR, such as the L858R missense mutation in exon 21, a deletion in exon 19, and the T790M mutation, demonstrated a higher degree of radiosensitivity than those containing wild‐type EGFR. This increased radiosensitivity manifested as overtly decreased cell viability, reduced clonogenic ability, and delayed DNA repair kinetics.^72^, ^73^ Nakamura et al. analyzed the failure pattern of definitive chemoradiotherapy in unresectable stage III non‐squamous NSCLC patients (*n* = 173, including 34 with active EGFR mutations and 13 with positive ALK rearrangement). Patients harboring EGFR mutations attained a dramatically improved 3‐year OS compared with those harboring wild‐type EGFR (75% vs. 46%, *p* = 0.002). Additionally, EGFRm cohorts experienced less in‐field relapse (*p* = 0.027). This might be explained by the fact that patients with EGFR mutations exhibit higher radiosensitivity, thereby achieving better disease control with the same chemoradiotherapy scheme, which is converted into longer OS.^74^

### 7.2 EGFR TKIs reduce radioresistance

Irradiation‐induced DNA damage includes single‐strand breaks (SSBs), double‐strand breaks (DSBs), and base damage through direct or indirect effects. Base damage and SSBs can be effectively repaired through base excision repair (BER) mechanisms,^75^ while DSBs are repaired primarily through two pathways: non‐homologous end joining (NHEJ) and homologous recombination (HR).^76^ Cell cycle arrest is an important component of the DNA damage response, facilitating DNA repair and the maintenance of genome stability.^77^

The EGFR signaling pathway is involved in ionizing irradiation‐induced DNA damage repair. First, EGFR directly regulates DNA repair. Ionizing irradiation induces EGFR heterodimerization, which results in the autophosphorylation of its intracellular kinase domain. Activated EGFR forms a complex with DNA‐PKcs in the cytoplasm and transports it into the nucleus. EGFR subsequently combines with DNA‐PKcs to participate in NHEJ‐mediated, binds to proliferating cell nuclear antigen and activates it,^78^ and binds to and phosphorylates ATM, thereby activating it.^79^ Second, signaling downstream of the EGFR is directly involved in DNA repair. RT activates the EGFR pathway, which contributes to the proliferation of cancer cells.^80^ Ionizing irradiation induces the activation of the EGFR/PI3K/AKT pathway. AKT forms a complex with DNA‐PKcs in the nucleus, promotes the recruitment of DNA‐PKcs to DSB sites, activates DNA‐PKcs, and participates in NHEJ repair. AKT also upregulates the expression of *MRE11* through the GSK3β/β‐catenin/LEF‐1 pathway, thereby also participating in HR‐mediated repair.^81^ Ionizing irradiation induces the activation of the EGFR/Ras/Raf/MEK/ERK pathway, activates XRCC1, PARP, and RAD51 proteins, and increases the expression of XRCC1 and PARP.^82^, ^83^, ^84^ XRCC1 repairs base damage and SSBs through BER; PARP assists DNA‐PKcs in NHEJ‐mediated repair; and RAD51 fine‐tunes HR‐mediated repair.

**FIGURE 3:**
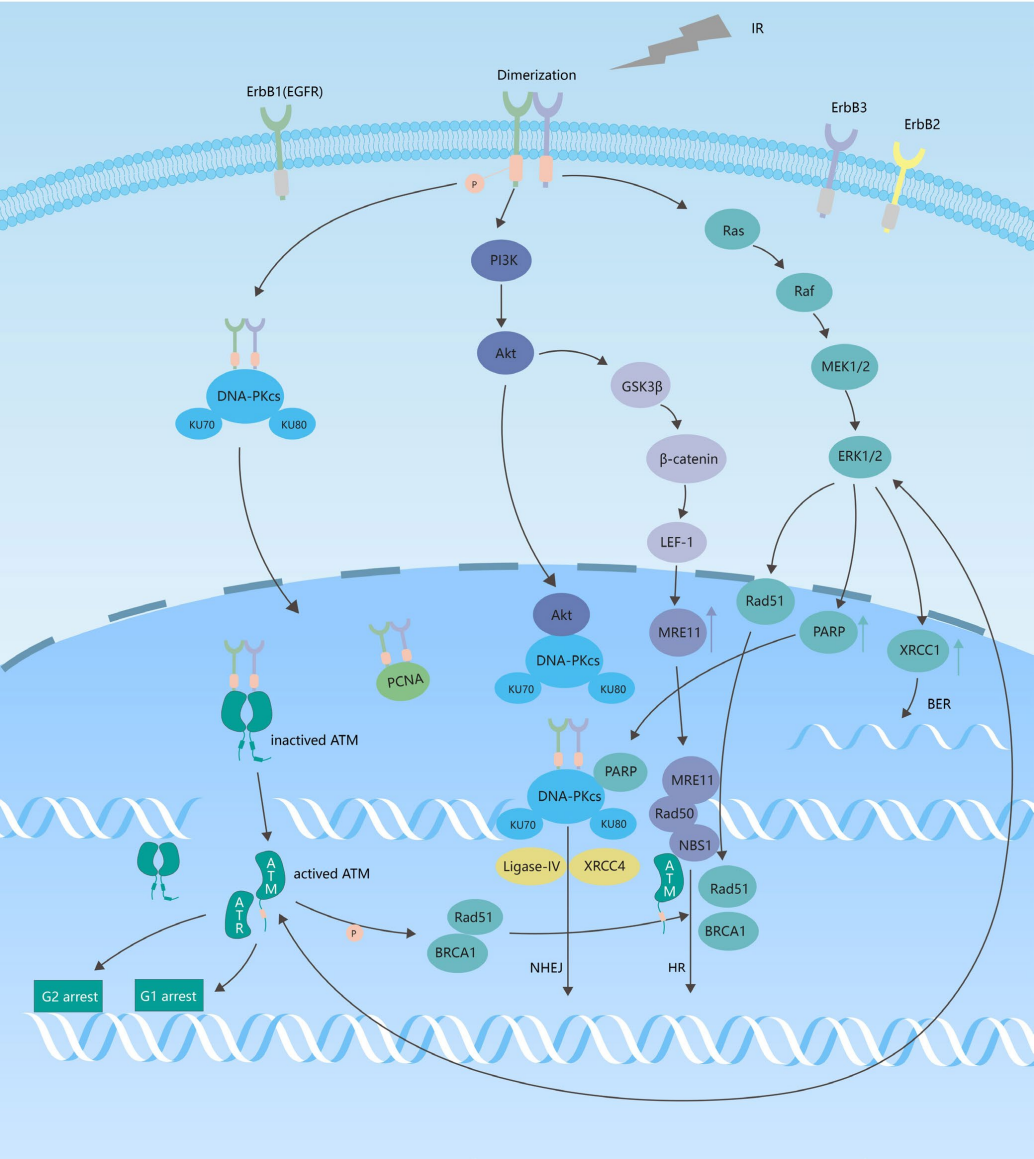
The EGFR signaling pathway is involved in irradiation (IR)‐induced DNA damage repair. ATM, ataxia telangiectasia mutation; ATR, ataxia‐telangiectasia and RAD3‐related; BER, base excision repair; DNA‐PKcs, DNA‐dependent protein kinase catalytic subunit; EGFR, epidermal growth factor receptor; ERK, extracellular‐regulated protein kinase; GSK3β; glycogen synthase kinase 3 beta; HR, homologous recombination; LEF‐1, lymphoid enhancer factor 1; MEK, mitogen‐activated extracellular signal‐regulated kinase; NHEJ, non‐homologous end joining; PARP, poly ADP‐ribose polymerase; PCNA, proliferating cell nuclear antigen; PI3K, phosphatidylinositol 3‐kinase; XRCC, x‐ray repair complementing defective repair in Chinese hamster cells

EGFR TKIs greatly inhibit RT‐induced DSB repair, thereby enhancing cancer cell radiosensitivity (Figure 3).^85^, ^86^

### 7.3 RT increases the TKI concentration in the CNS and reduces the probability of T790M occurrence

WBRT and/or local RT can disrupt the BBB to a certain extent, resulting in increased permeability to TKIs.^87^, ^88^, ^89^

The T790M mutation is responsible for more than 50% of the secondary resistance to EGFR TKIs, an effect that can be reduced by irradiation.^90^ In a clone formation assay using the H1975 and H3255 cell lines (double‐mutant [L858R plus T790M] and single‐mutant [L858R] for EGFR, respectively), Li et al. found that the SF2 value for H1975 and H3255 was 0.62 and 0.64, respectively, suggesting that the T790M mutation does not affect the radiosensitivity of NSCLC cell lines. Without x‐ray irradiation, the IC50/H1975 to IC50/H3255 ratio following gefitinib treatment was 85.9; however, this ratio decreased markedly to 39.2 after irradiation with 2.5 Gy.^90^

## 8 POSSIBILITY OF CONTINUED TKI BEYOND OLIGO‐PROGRESSION

After disease progression on first‐line EGFR‐TKI, disease flare may occur when TKI is abruptly discontinued, including aggravation of symptoms, an increase in tumor size, and increased FDG uptake on PET‐CT.^91^ Therefore, it has been suggested that TKI should be continued after progression in selected clinical situation in patients who have previously responded to EGFR inhibitors, which has been verified in many retrospective studies.^92^

In 2015, the ASPIRATION, a phase II, single‐arm study was conducted to study the efficacy of first‐line erlotinib therapy and post‐progression erlotinib therapy in patients with stage IV and EGFR mutation‐positive NSCLC. Of 207 intent‐to‐treat patients, 176 had a PFS1 event (171 progression and 5 deaths); of these, 93 continued erlotinib therapy following progression, while 78 discontinued. Median PFS1 (time to Response Evaluation Criteria in Solid Tumors 1.1 progression or death) was 10.8 months. In the 93 continuing patients, median PFS1 was 11.0 months and PFS2 (time to off‐erlotinib progression if erlotinib therapy was extended beyond progression at patient and/or investigator discretion) was 14.1 months. The study supported that continuing treatment of erlotinib beyond RECIST PD is feasible in selected patients such as slow PD.^93^ However, the other randomized phase III IMPRESS trial in the same year which compared continuation of gefitinib plus platinum‐based doublet chemotherapy with switching to chemotherapy alone did not achieve positive results. PFS in the two groups has no difference, all of which was 5.4 months.^94^ Hence, it seems controversial whether this drug is still useful after first‐line TKI progression, and it may be beneficial in certain populations, such as those with slow progression, asymptomatic, and good ECOG/PS scores.

In the past few years, scholars have realized that the PD of NSCLC with EGFRm is heterogeneous as well, including systemic PD, oligo‐PD, and CNS sanctuary PD.^95^ Oligo‐PD represents an inert state which targeted therapy has resulted in either stable disease or a partial/complete response with progression only in limited number of sites. In this situation, a majority of diseases may continue to be controlled by previous TKI, and adding hypo‐fractionated image‐guided radiation therapy or SBRT may maintain patients on their current systemic regimen, delay the time to change in therapy, and prolong the time to the second progression, while ablating the few progressive metastases. For example, in 206 patients with stage IIIB/IV EGFRm NSCLC who had oligo‐PD during the first‐line EGFR‐TKI therapy, continuation of TKI with addition of LAT demonstrates a PFS1 of 10.7 months, PFS2 of 18.3 months, 1‐, 2‐, and 3‐year survival rates of 94.1%, 78.9%, and 54.7%, respectively. Local ablation for the oligo‐progressive lesions with continuous EGFR‐TKI treatment is associated with additional 18.3 months of disease control.^96^ Similarly, in another cohort of 18 patients treated with local therapy plus continued EGFR‐TKI therapy, the median time to the second progression was 10 months, median time to change treatment regime was 22 months.^97^

**FIGURE 4:**
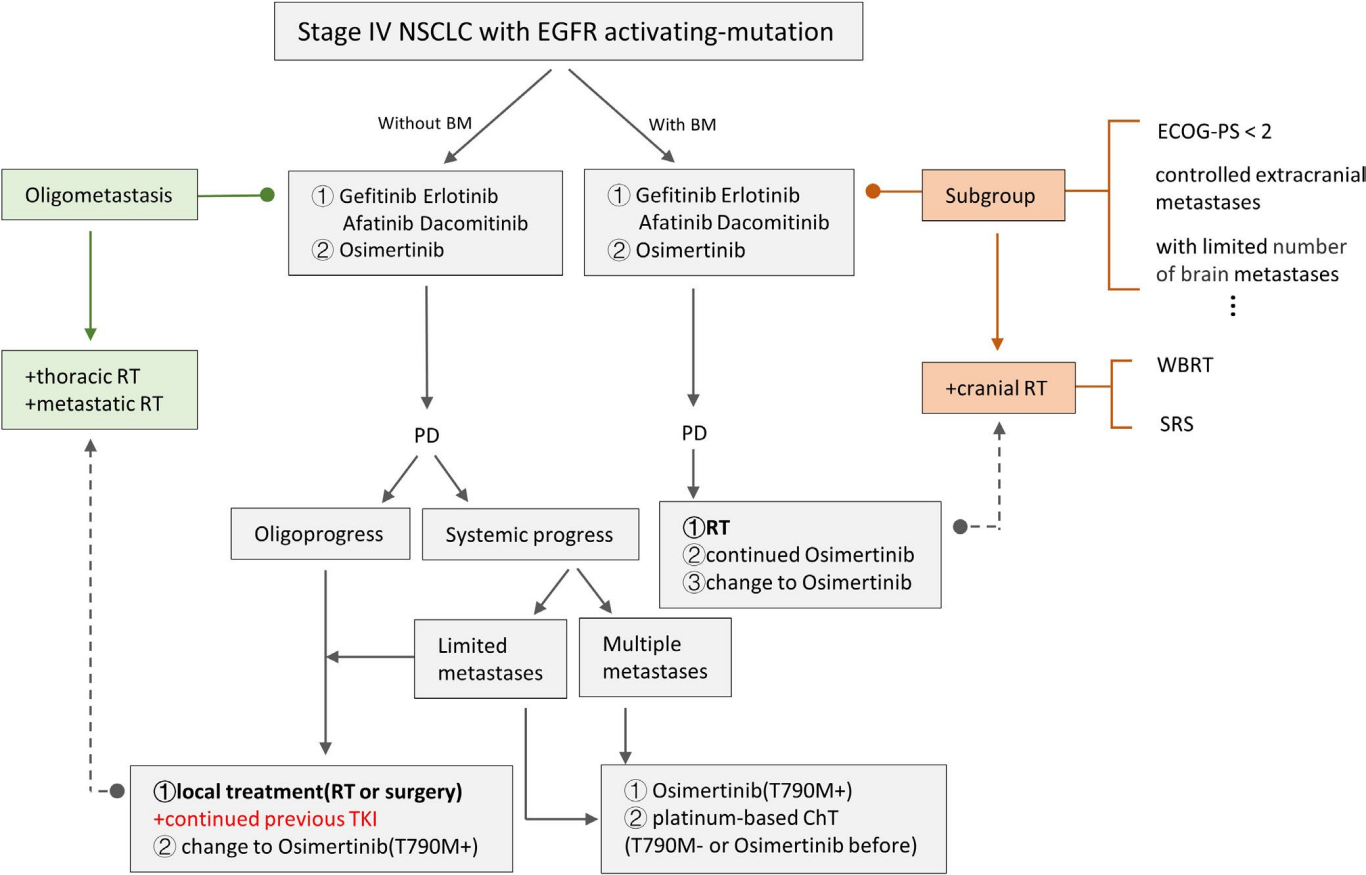
Flow diagram with therapeutic options in EGFR‐mutated stage IV NSCLC. BM, brain metastasis; ChT, chemotherapy; CR, complete remission; EGFR, epidermal growth factor receptor; NGS, next‐generation sequencing; NSCLC, non‐small cell lung cancer; PD, progressive disease; PR, partial remission; RP, radiation pneumonitis; RT, radiotherapy; SD, stable disease; SRS, stereotactic radiosurgery; TKI, tyrosine kinase inhibitor; TRT, thoracic radiotherapy; WBRT, whole‐brain radiotherapy

ESMO clinical practice guidelines for metastatic NSCLC in 2018 and Canadian consensus in 2019 recommended that continuous use of current targeted therapy in combination with local treatment could be considered as an approach in certain selected patients with limited extra‐CNS oligo‐PD in EGFR‐mutation positive NSCLC.^98^, ^99^ NCCN guideline in 2021.v2 also suggested continual EGFR‐TKI with or without local therapy in EGFRm advanced/metastatic NSCLC patients with asymptomatic PD and symptomatic limited PD after first‐line erlotinib, afatinib, gefitinib, or osimertinib therapy, except for the occurrence of T790M. Overall, compared with systemic progression, it is possible to consider continuing EGFR‐TKI treatment after oligo‐PD, especially in combination with LAT (Figure 4).

## 9 CONCLUDING REMARKS

This review systemically explored the role of RT in EGFRm stage IV NSCLC. For the oligometastatic setting, RT to both primary and metastatic lesions might prolong both PFS and OS. Reduced‐dose TRT and critical restriction of the MLD before TKI resistance onset hold some promise. Although EGFR TKIs can exert synergistic effects with RT, this regimen should be applied with caution owing to the risk of adverse effects, especially lung damage. In the first‐ or second‐generation EGFR TKI era, upfront brain RT might achieve better DCRs and iPFS, even in asymptomatic populations; however, this is expected to change with the advent of next‐generation TKIs with increased CNS efficacy. The likely beneficiaries of upfront brain RT, especially SRS, with the arrival of the new drugs should be identified through clinical trials. We have provided a flow diagram depicting the therapeutic options, including RT, for the treatment of EGFRm stage IV NSCLC (Figure 4), which is expected to be of considerable help in clinical practice.

## CONFLICT OF INTEREST

The authors made no disclosures.

## ETHICS APPROVAL AND CONSENT TO PARTICIPATE

This is a review, and this article does not contain any studies with human participants or animals.

## Data Availability

All data generated or analyzed during this study are included in this published article.
